# Drivers of Daily Routines in an Ectothermic Marine Predator: Hunt Warm, Rest Warmer?

**DOI:** 10.1371/journal.pone.0127807

**Published:** 2015-06-10

**Authors:** Yannis P. Papastamatiou, Yuuki Y. Watanabe, Darcy Bradley, Laura E. Dee, Kevin Weng, Christopher G. Lowe, Jennifer E. Caselle

**Affiliations:** 1 School of Biology, Scottish Oceans Institute, University of St Andrews, St Andrews, Scotland, United Kingdom; 2 National Institute of Polar Research, Tachikawa, Tokyo, Japan; 3 SOKENDAI (The Graduate University for Advanced Studies), Tachikawa, Tokyo, Japan; 4 Bren School of Environmental Science and Management, University of California Santa Barbara, Santa Barbara, California, United States of America; 5 Department of Fisheries Science, Virginia Institute of Marine Science, Gloucester Point, Virginia, United States of America; 6 Department of Biological Sciences, California State University Long Beach, Long Beach, California, United States of America; 7 Marine Science Institute, University of California Santa Barbara, Santa Barbara, California, United States of America; Aristotle University of Thessaloniki, GREECE

## Abstract

Animal daily routines represent a compromise between maximizing foraging success and optimizing physiological performance, while minimizing the risk of predation. For ectothermic predators, ambient temperature may also influence daily routines through its effects on physiological performance. Temperatures can fluctuate significantly over the diel cycle and ectotherms may synchronize behaviour to match thermal regimes in order to optimize fitness. We used bio-logging to quantify activity and body temperature of blacktip reef sharks (*Carcharhinus melanopterus*) at a tropical atoll. Behavioural observations were used to concurrently measure bite rates in herbivorous reef fishes, as an index of activity for potential diurnal prey. Sharks showed early evening peaks in activity, particularly during ebbing high tides, while body temperatures peaked several hours prior to the period of maximal activity. Herbivores also displayed peaks in activity several hours earlier than the peaks in shark activity. Sharks appeared to be least active while their body temperatures were highest and most active while temperatures were cooling, although we hypothesize that due to thermal inertia they were still warmer than their smaller prey during this period. Sharks may be most active during early evening periods as they have a sensory advantage under low light conditions and/or a thermal advantage over cooler prey. Sharks swam into shallow water during daytime low tide periods potentially to warm up and increase rates of digestion before the nocturnal activity period, which may be a strategy to maximize ingestion rates. “Hunt warm, rest warmer” may help explain the early evening activity seen in other ectothermic predators.

## Introduction

Animals must make decisions regarding when and how intensely to vary rates of activity. The precise function of increased activity will change over different time frames, but over shorter time scales is likely related to foraging or predator avoidance. For larger predators, safety may not be the dominant driver of activity, but the animal must still decide when and how intensely to forage. As a predator becomes more active, its chance of locating prey will increase. However, there is a metabolic cost associated with increased activity: the higher the intensity of foraging, the higher the potential for body condition to decrease, especially if the predator is not successful in capturing prey [1]. This compromise between increased chance of foraging success and loss of body condition may lead to daily routines over a diel cycle, with peaks in activity followed by resting periods [1].

Many predators, in both terrestrial and marine systems, display peaks in activity during crepuscular periods particularly during the early evening [2–6]. The timing of these peaks is thought to coincide with periods of increased nocturnal foraging success, but in some cases may also be related to predator avoidance [5]. However, with ectothermic predators it is also important to consider environmental changes in temperature as thermal effects may play a strong role in an animal’s daily routines [7, 8]. Ectotherms are intimately connected to changes in environmental temperature, from the molecular and cellular levels (e.g. enzyme kinematics, metabolic rates) to the individual and population levels (e.g. rates of digestion, predator functional response, trophic interactions; [9, 10]). In general, the influence of temperature on behavioural or physiological performance can be described by a thermal performance curve [11]. Peak performance occurs at an optimal temperature (T_opt_), with temperatures < T_opt_ causing a gradual decline in performance (described by a Gaussian function), and temperatures > T_opt_ leading to a rapid decline (quadratic function) until some critical temperature is reached [11]. Therefore, species occupy a thermal niche bounded by critical temperatures within which performance may vary widely and non-linearly.

Over diel time frames, temperature changes can be significant and ectotherms may modify behaviours to take advantage of thermal regimes. Two behavioural routines have been proposed for marine predators: ‘hunt warm, rest cold’, and ‘hunt cold, rest warm’ [12]. With ‘hunt warm, rest cold’ foraging occurs in habitats or time periods of higher temperatures, elevating metabolic rates and improving foraging efficiency [7]. Energy expenditure is then minimized by resting in cooler waters, thereby reducing metabolic rates. A reduction in body temperature may cause an increase in digestive efficiency by reducing overall rates of digestion, exposing prey to digestive enzymes for longer periods of time [13]. Alternatively, ‘hunt cold, rest warm’ describes the case of an animal resting in warmer water, which appears counterproductive as energy expenditure will increase. However, rates of gastric evacuation will also increase, leading to a quicker return of appetite thereby potentially maximizing feeding rates [12, 14, 15]. It is likely that the optimal behavioural routine is dependent on prey abundance. If prey is abundant then we may expect the predators to maximize feeding rates (i.e. hunt cold, rest warm), while in low resource environments it may be more advantageous to maximize the efficiency of energy extraction (i.e. ‘hunt warm, rest cold’, see [12]).

Many large carnivores are ‘digestion-limited’ in that they spend more time digesting than they do searching for food [16]. Furthermore, the increased metabolic rate associated with digestion (‘specific dynamic action’) can reduce the activity of the animal, as the animal will be closer to the upper limit of its metabolic scope (e.g. [17]). Therefore, it is important to consider the role digestion plays in animal movements, and how temperature can influence it. Finally, the daily routines of predators will also be related to the behaviour of potential prey, and temperature can have a significant influence on the relationship between the two. There may be asymmetries in the thermal response of consumer-resource dynamics, due to variation in body velocities or prey escape responses and these can qualitatively and quantitatively change predator-prey dynamics [8, 10]. Testing these ideas in the field requires an understanding of both predator and prey activity cycles and the role of temperature in driving them.

Clearly there can be many potential drivers of daily activity cycles and disentangling them is difficult, particularly for marine predators where visual observations of behaviour are difficult or impossible. Many species of shark are upper level predators and the timing of their activity has ecological and conservation consequences, as they can directly influence the daily routines and habitat selection of lower trophic levels [18]. The majority of sharks are thought to be nocturnal hunters although this assumption often comes from fishing data or horizontal and vertical movement data, all of which can make interpretation of activity difficult [2, 19]. However, the development of animal-borne sensors that directly measure activity and behaviour now allows hypotheses regarding predator behaviour to be tested in the field [2, 20, 21, 22].

The blacktip reef shark (*Carcharhinus melanopterus*, Quoy and Gaimard 1824) is found in shallow reef and coastal habitats in tropical ecosystems of the Pacific and Indian Oceans [23, 24]. Adult blacktip reef sharks must swim continuously in order to extract enough oxygen from seawater to meet metabolic demands, and therefore never truly rest. They use relatively small home ranges and in some locations females have been hypothesized to behaviourally thermoregulate, swimming into warm, shallow water during the day, presumably to increase body temperatures [23, 24]. Visual observations of blacktip reef sharks while they were in shallow water suggested that sharks were not foraging, although activity was not specifically recorded [24]. We used bio-logging to quantify the daily activity cycles, and assess the drivers of these cycles, for blacktip reef sharks at Palmyra, a remote Pacific atoll. We simultaneously used behavioural observations of focal herbivorous fishes to quantify activity cycles of potential diurnal prey. Blacktip reef sharks will have relatively slow rates of digestion, but those at Palmyra reside in a location where prey abundance is high [25, 26]. We therefore predict that these reef sharks use a ‘hunt cold, rest warm’ strategy, to maximize ingestion rates [23, 25]. Our specific predictions are that a) blacktips are most active during early morning periods when they are cooler, b) sharks are least active during late afternoon when temperatures are high, and c) rates of digestion are highest during periods when sharks are least active which also coincides with warmer body temperatures.

## Materials and Methods

### Ethical standards

Tagging experiments were approved by the University of California Santa Barbara IACUC (#856), and University of Hawaii IACUC (# 03-66-3). All research carried out at Palmyra atoll was approved by US Fish and Wildlife service (USFWS).

### Study site

**Fig 1:**
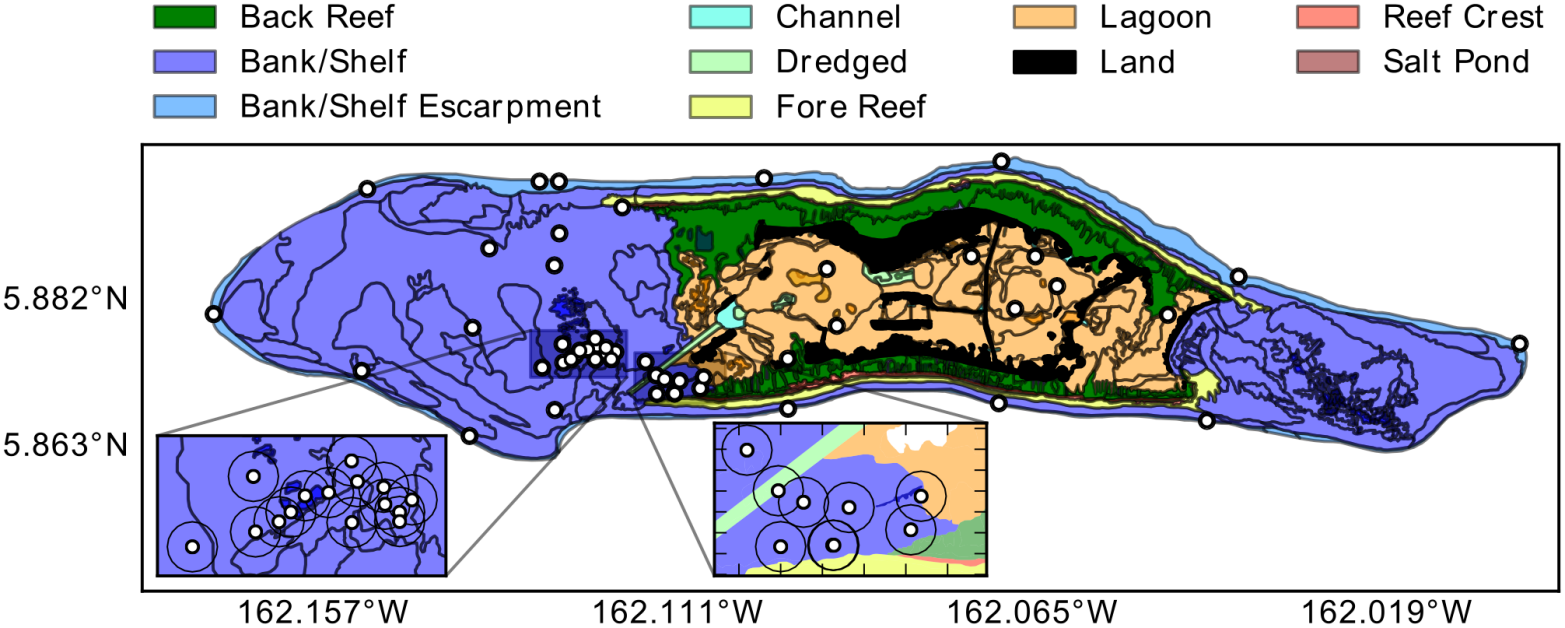
Map of Palmyra Atoll showing different habitat types and the location of acoustic listening stations (yellow circles). Black circles around receivers shows approximate detection radius.

Palmyra atoll (5°54’ N; 162°05’ W) is a US National Fish and Wildlife Refuge located just north of the equator in the central Pacific Ocean (Fig 1). Due to bans on fishing, large numbers of predators (sharks, snapper, and trevally) are found at the atoll [26]. Habitat types consist of deep murky lagoons (50 m), surrounded by sandflats which are exposed to air during extreme low tides. These connect to backreefs (2–3 m depth, high vertical relief coral, good visibility) which transition to forereefs consisting of a steep slope with high coral cover and high water clarity (Fig 1, for more details see [23, 26]). All research conducted at Palmyra was approved by USFWS and The Nature Conservancy.

### Telemetry and data-loggers

A combination of acoustic telemetry and animal-borne data-loggers were used to quantify shark activity, body temperature, and habitat use. Transmitters measure parameters over periods of months to years, but the temporal resolution is low (minutes to hours or days). Acoustic transmitters consisted of a mix of V16, V13 and V9 types (69 kHz, Vemco Ltd., Nova Scotia, Canada). Transmitters were coupled with sensors allowing remote measurements of behavioural and physiological data. Blacktip reef sharks were caught on the backreefs using hook and line and restrained alongside the boat. We inverted sharks on their backs inducing tonic immobility, a natural trance-like state (e.g. [23]). Six sharks were tagged with V13AP or V9AP (42x13 mm 12.2 g, 43x9 mm 6.1g) transmitters which measure acceleration and swimming depth. The acceleration transmitters measure body acceleration along 3-axes (at 5 Hz), filter out the static contribution due to gravity, and over a 17 second interval calculate a root mean square (RMS) acceleration value from the 3 axes combined, which is then transmitted. There is a delay of 20–35 seconds between transmissions. Acceleration sensors should be stationary relative to the animal’s body axis, so we externally attached AP transmitters to the dorsal fin (to avoid rolling inside the body cavity). To quantify changes in body temperature, four of these six individuals also had a V16T (54x16 mm, 19 g) transmitter surgically implanted into the body cavity (resolution 0.1°C, Table 1), via a 2–3 cm incision in the ventral surface. The incision was made through the dermal and outer muscle layer, so that the transmitter was residing inside the body cavity adjacent to the viscera. A single uninterrupted suture was used to close the wound and the animal was measured, sexed, and released. Total time between capture and release was less than 10 min. A network of 65 underwater omnidirectional acoustic receivers (VR2W, Vemco Ltd.) are maintained throughout Palmyra (all habitat types), which listen for transmitter equipped sharks (Fig 1). Each time a transmitter equipped shark swims within range (range varies by habitat type from 200 m in backreefs to 500 m in lagoons and forereefs, [27]) of an acoustic receiver, the time, date, and sensor values (i.e. depth, acceleration, body temperature) are recorded, along with the unique transmitter code so that individual animals can be identified. We retrieved, downloaded and re-batteried listening stations annually. All sharks were tagged in July and August of 2011 and 2012.

**Table 1:** Blacktip reef sharks were fitted with acoustic transmitters or data-loggers.

Individual	Tagging date	Total Length (cm)	Transmitter/Data-logger type	Duration detected (days)
1	2/8/11	116	V9AP	35
2	2/8/11	118	V9AP	23
3	21/8/12	122	V13AP, V16T	305
4	21/8/12	133	V13AP, V16T	295
5	24/8/12	127	V13AP, V16T	307
6	24/8/12	105	V13AP, V16T	352
7	4/7/13	123	ADT	4
8	10/7/13	117	ADT	4
9	15/7/13	113	ADTS	4
10	19/7/13	127	ADTSV	4
11	23/9/07	110	G	21

Vemco acoustic tags are designated ‘V’. VAP are acceleration/depth sensing acoustic transmitters, VT are temperature sensing acoustic transmitters. ADT are data-loggers with 3-axes acceleration, depth and temperature sensors. ADTS additionally include speed sensors, and ADTSV also includes a video camera. G is a gastric motility data-logger. All sharks were female.

To obtain higher temporal resolution behavioural data, four additional sharks were fitted with data-logger packages. Data-loggers are attached to sharks for periods of several days, and record continuously and at high frequency, but must be recovered in order for data to be obtained (unlike transmitters). Two sharks were fitted with ORI400-D3GT loggers (12-mm diameter, 45-mm length, and 9 g; Little Leonardo Co., Tokyo, Japan) which had sensors that recorded 3D acceleration (20 Hz), depth and water temperature (sample rate 1 sec^-1^), while an additional two sharks were fitted with W190L-PD3GT loggers (21-mm diameter, 117-mm length, and 60 g; Little Leonardo Co.) which also record swim speed (sample rate 1 sec^-1^). In order to record specific behaviours and habitat use, we fitted one shark with a Little Leonardo DVL400 video camera (23 x 112 mm, 80 g, recording duration 6 h), which recorded at 640 x 480 pixels at 30 frames/second. To avoid filming during periods of potential stress associated with capture, the camera was programmed to begin recording 24 h after the animal was released. All sensors were embedded in syntactic foam floats and fitted to the dorsal fin via a tie-wrap through two small punctures in the fin (holes were small as only the flattened tie-wrap had to fit through). Four days after the animal was released, a timer caused the tie-wrap to break and the entire package floated to the surface [20]. The data-logger packages were then located and retrieved via an embedded VHF transmitter. A couple of individuals that had been fitted with data-logger packages were re-sighted a year later with no markings or wounds of any sort on the dorsal fins.

We calculated Overall Dynamic Body Acceleration (ODBA) from 3D acceleration data as a metric of activity. ODBA was calculated by removing the static contribution of gravity from acceleration data using a high-pass filter, and then combining the absolute values of acceleration from all three axes [28, 29]. ODBA correlates well with oxygen consumption and energy expenditure in a wide range of animals, including sharks [28, 29]. We did not aim to calculate absolute energy expenditure but we felt that ODBA provided a reasonable overall measure of activity.

Quantifying when rates of digestion are highest provides further insights into the drivers of diel behaviour. The stomach is a muscular organ that mixes prey items with digestive fluids and transfers digested products from the stomach to the intestine. Hence, real-time measurements of gastric motility provide insight into the state of digestion [25, 30]. We recorded motility using an inflection sensor coupled to a data-logger (140 mm length, 19 mm diameter, mass in air 45 g, earth & Ocean Technologies, Kiel, Germany). The sensor consists of a piezoelectric film which generates a voltage every time the film flexes, with the data-logger sampling a single value every 15 seconds, based on the number and speed of inflections [30]. The data-logger was attached to a V13 acoustic pinger and force fed to a single blacktip reef shark at Palmyra in September 2008. The shark retained the logger for 21 days then regurgitated it, after which we located the device using a diver held underwater hydrophone. There appeared to be logger failure one week after deployment, so we only analysed data from the first seven days of deployment. We periodically located the shark during the deployment period and snorkelers visually observed its behaviour, with no evidence of stress or unusual behaviours. Previous captive experiments showed that blacktip reef sharks will resume feeding within 24 h of logger deployment [25]. The first 24 h of data were discarded. Estimated mass of all sharks was 7–10 kg so tag: body mass ratios were all below 1.4%, which has been suggested as a cut-off for other predatory fishes [31].

### Potential prey activity

The diet of blacktip reef sharks has not been well quantified but will include a wide range of teleosts [32]. As a metric of potential prey activity, divers followed common herbivorous fishes on Palmyra’s backreefs and recorded foraging activity (bite rates) defined as the number of bites per minute. These observations only provide information on diurnal prey species as we were limited by safety regulations that prohibit diving at night. Three species were tracked: *Acanthurus nigricans*, *Ctenochaetus striatus*, and *Acanthurus lineatus*. During observations, the diver stayed a significant distance from the focal species so as not to disturb its behaviour. For each focal individual, all bites were recorded during a 5-minute interval to determine a bite rate. Follows were conducted throughout the day in the same backreef sites as shark tagging, simultaneously with the period of data-logger deployments.

### Data analysis

Sensor data were analysed using generalized additive mixed models (GAMMs), which allow for both the specification of serial auto-correlation and the inclusion of shark identity as a random effect to account for differences between individuals. For all analyses, we sub-sampled telemetry data with one-hour mean values, while data from loggers were sampled over one minute means. We used an AR1 function with time as the position variable to account for serial correlation in time series data. The AR1 function also calculates the magnitude of serial dependence as a function of distance between time steps; the correlation at lag = 1 was then included as a term in the model to specify the correlation structure. All GAMMs were constructed with a Gaussian error distribution, and all covariates were modelled with smooth splines (with the exception of month in the acceleration transmitters which was included as a control). We were primarily interested in how sensor variables (ODBA, acceleration, swimming depth, body temperature, gastric motility) varied with time of day and tidal or lunar cycle, which were modelled with cyclic smoothing splines. ODBA and speed were modelled controlling for depth (to isolate tide effects). Data-logger deployment was concurrent with the installation of a tidal station, providing high resolution data on tidal height. Tide data was not available during transmitter deployments so instead we modelled lunar cycle as a covariate. Lunar phase was included as % illumination and was downloaded from the United States Naval Observatory Astronomical Applications Department (http://aa.usno.navy.mil/data/docs/MoonFraction.php). Although we cannot directly translate lunar phase into tidal cycle, high and low values of percentage illumination indicate extreme tidal highs and lows. To remove seasonal effects, and because we were interested in behaviour over diel time scales, we only analysed body temperature data from July-November. All statistics were generated in R (R Development Core team 2013) using the *mgcv* package [33] and models were selected using Akaike Information Criteria, Bayesian Information Criteria, and Maximum Likelihood Estimates. We combined animals for analysis, but due to small sample sizes we also analysed data for individuals separately (Online Resources).

## Results

### Telemetry and data-loggers

**Fig 2:**
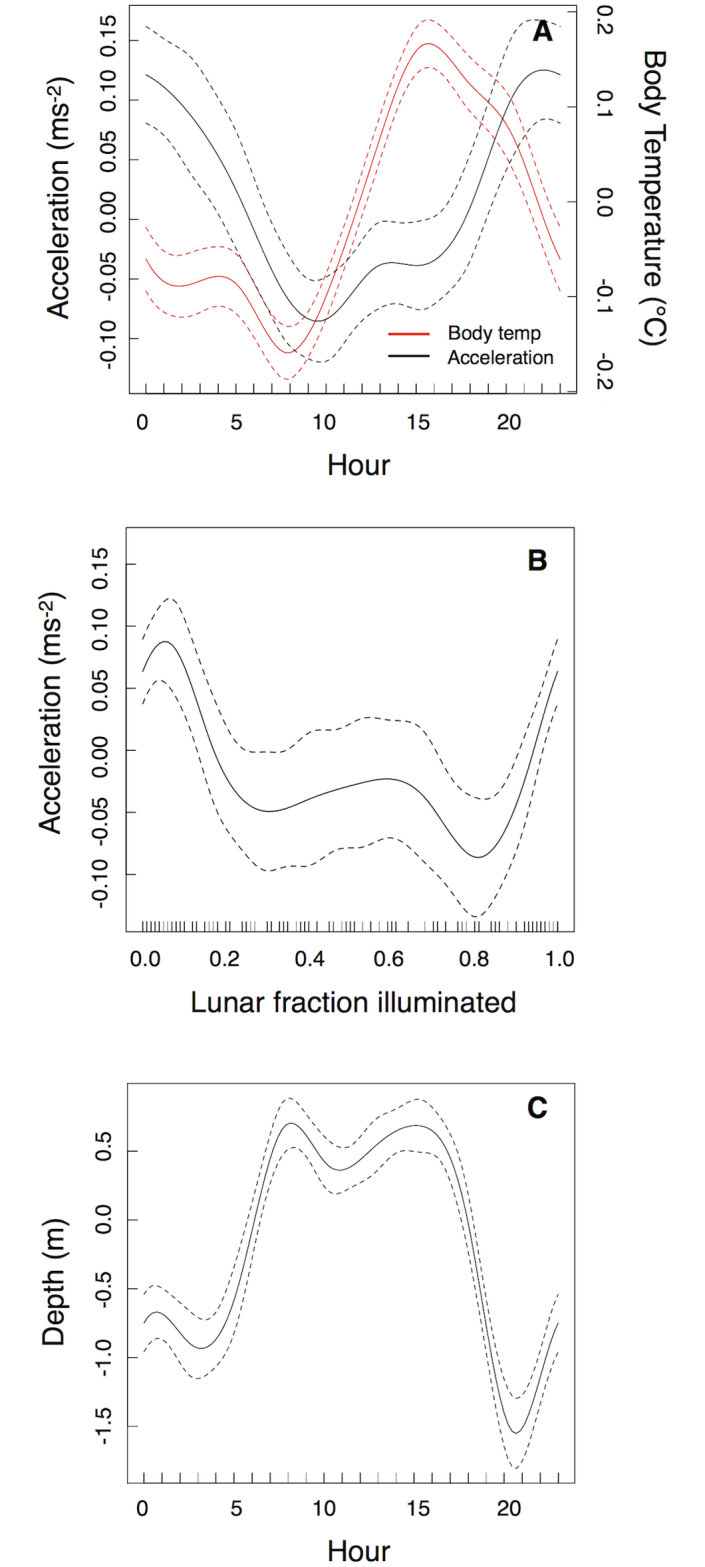
Effects of time of day and lunar cycle on shark activity, swimming depth and body temperature. Results of generalized additive mixed models (GAMMs) for telemetry data from blacktip reef sharks. Shown are diel changes in acceleration and body temperature (A), change in activity with lunar cycle (B) and diel changes in swimming depth (C). Dashed lines indicate the 95% confidence interval around each smooth term. Y-axis represents the standardized residuals.

Telemetry data were collected over a two-month period with sharks showing clear patterns of activity and swimming depth associated with diel and lunar cycles (Fig 2, Table 2, S1A Table). Acceleration levels were highest at night and during the periods of both the new and full moons (Fig 2A and 2B). Sharks used deeper water during the day when they were least active and shallower water at night (range of depth change was only 2–4 m, Fig 2C, Table 2). There were clear patterns in body temperature, with highest temperatures in the late afternoon (approximately 15:00–16:00) and lowest values occurring in the morning (5:00–10:00, body temperature varied by 2.2–2.7°C, Fig 2A, S1A and S1D Table, S1D–S1F Fig). The peak in activity recorded by the transmitters lagged several hours behind the peak in body temperature.

**Table 2:** GAMM results showing effects of time of day, tides or lunar cycle on body temperature, acceleration and swimming depth (from telemetry), and ODBA and gastric motility (from data-loggers).

Model and terms x	*n*	Smoother	Degrees of freedom	*F*-statistic	*P*	*r* ^*2*^
Body temperature (*telemetry*)	4					0.09
s(Hour)		cyclic	7.15	32.14	<0.001	
Gastric motility (*data-logger*)	1					0.05
s(Hour)		cyclic	6.20	18.82	<0.001	
Acceleration (*telemetry*)	6					0.13
s(Hour)		cyclic	4.45	13.97	<0.001	
s(Lunar fraction illuminated)		cyclic	5.00	7.83	<0.001	
factor(Month)		—	—	—	<0.001	
Depth (*telemetry*)	6					0.16
s(Hour)		cyclic	7.65	42.39	<0.001	
ln ODBA (*data-logger*)	4					0.08
s(Time of Day*)		cyclic	6.80	25.45	<0.001	
s(Tide)		cubic	6.86	18.14	<0.001	
s(Ambient Temperature)		cubic	5.57	5.57	<0.001	
s(Depth)		cubic	6.30	6.30	<0.001	

Only significant effects are shown for models with the lowest AIC and BIC values. Models differed by > 2 AIC units from the next possible model to be selected. Results are for all individuals combined.

**Fig 3:**
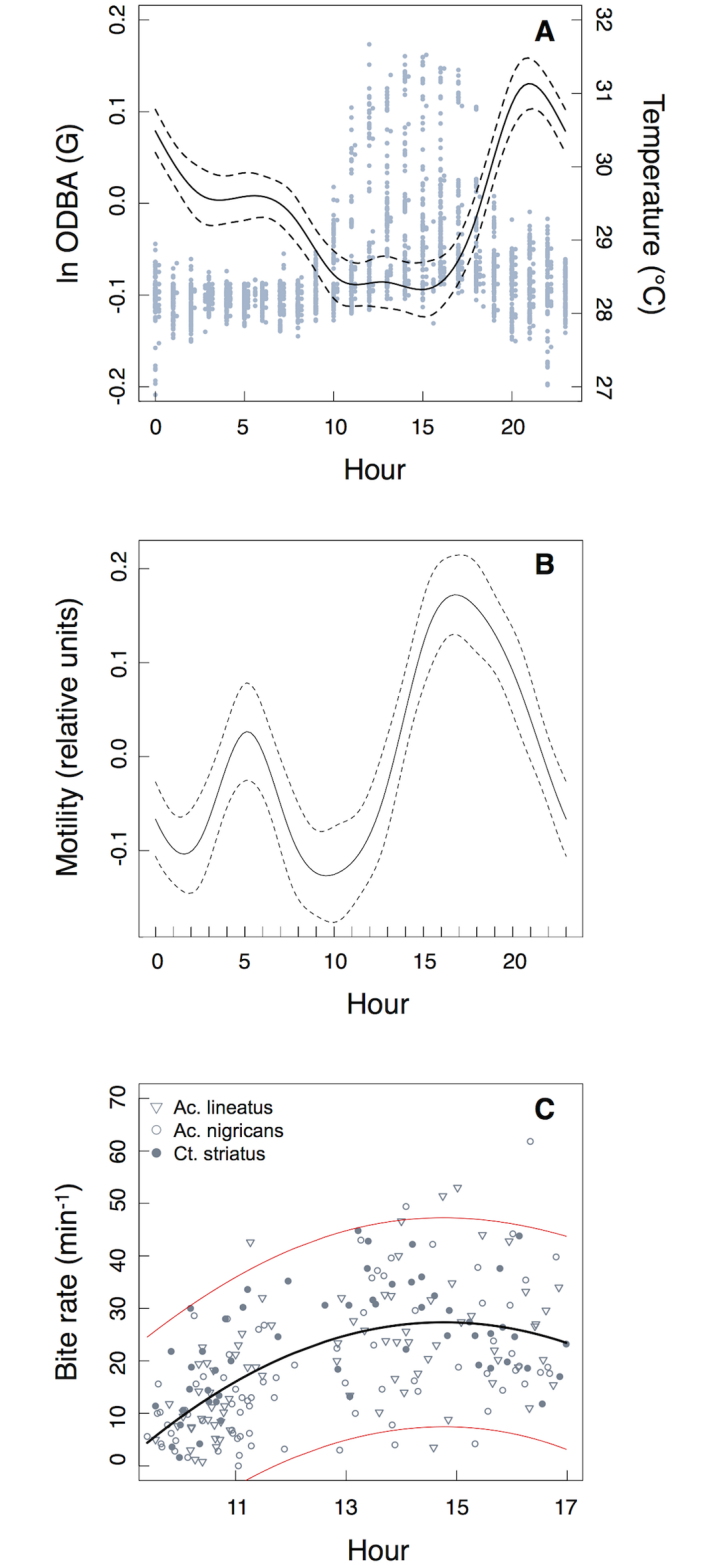
Effects of time of day on predator and prey activity. A) GAMM results for changes in Overall Dynamic Body Acceleration (ODBA) as a function of time of day; water temperature (from the data-loggers) has been overlaid (blue points). B) GAM results for change in gastric motility as a function of time of day from a single blacktip reef shark. Dashed lines indicate the 95% confidence interval around each smooth term. Y-axis represents the standardized residuals. C) Foraging rates for three species of herbivorous reef fish, from Palmyra’s backreefs *(Acanthurus lineatus*, *A*. *nigricans*, *Ctenochaetus striatus*). A quadratic regression has been fit to the bite rate data (with 95% confidence intervals shown in red).

Over the shorter time period measured by the data-loggers, all sharks demonstrated a peak in activity from 19:00–21:00, and for three of those four sharks this coincided with the ebbing tide (Fig 3A, Table 2, S1B Table, S1A–S1C Fig). Activity generally remained high throughout the evening and lowest levels of activity occurred between 10:00–15:00. ODBA also varied over the tidal cycle being highest at times of high (but ebbing) tides (Table 2, S1C Fig). In all cases, peaks in ODBA lagged behind the peak in ambient water temperature by 3–5 h (Fig 3A). Lowest values of ODBA occurred when the sharks were in the warmest water. Water temperature over the diel cycle varied by 3–4°C.

**Fig 4:**
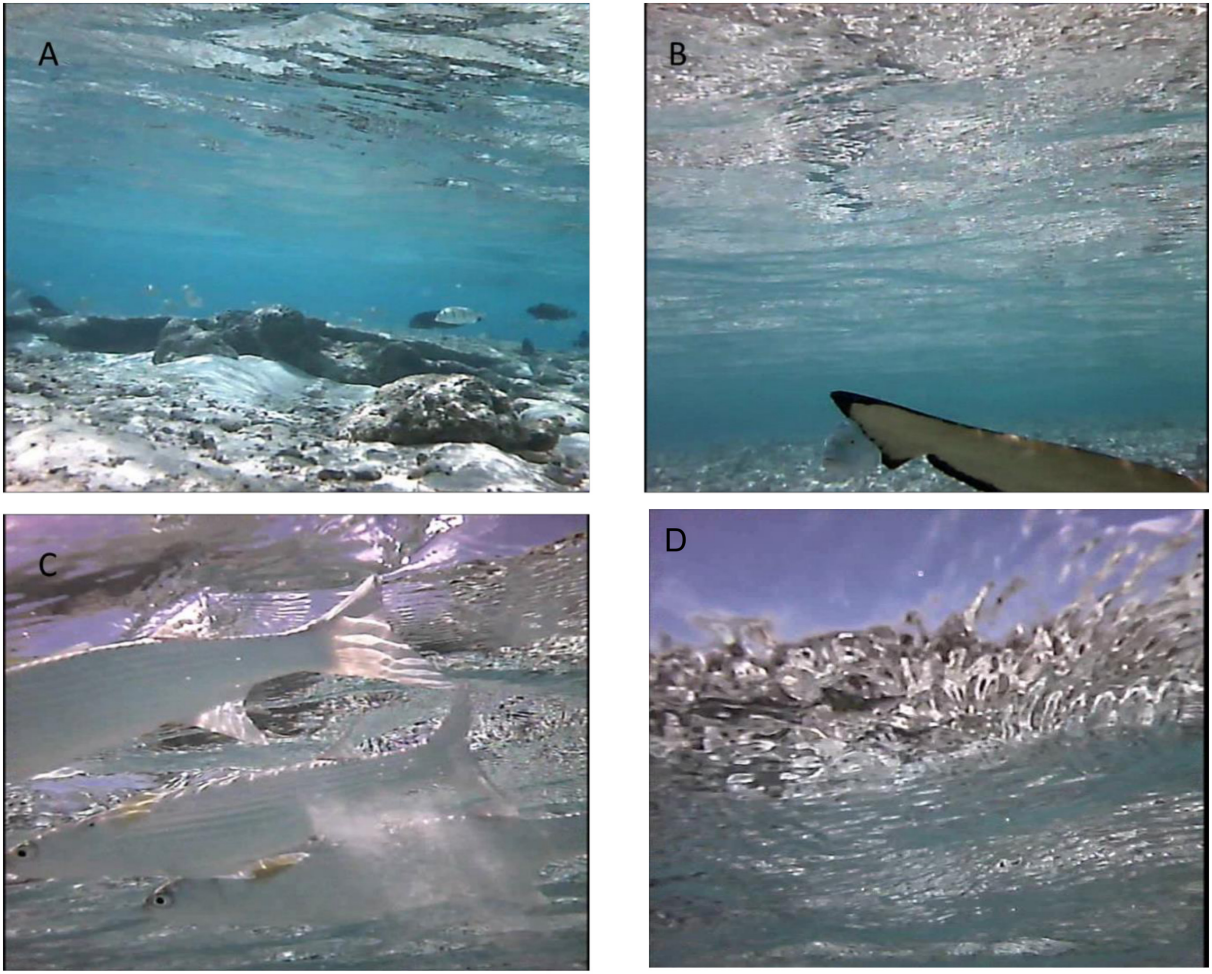
Images from video camera attached to a blacktip reef shark. Footage was taken during low tide in the morning and shows the shark using sandflat and inner backreef habitats (A), closely following another blacktip while a giant trevally (*Caranx ignobilis*) investigates (B), and frequently swimming up into school of mullet (*Mugil cephalus*, C).

**Fig 5:**
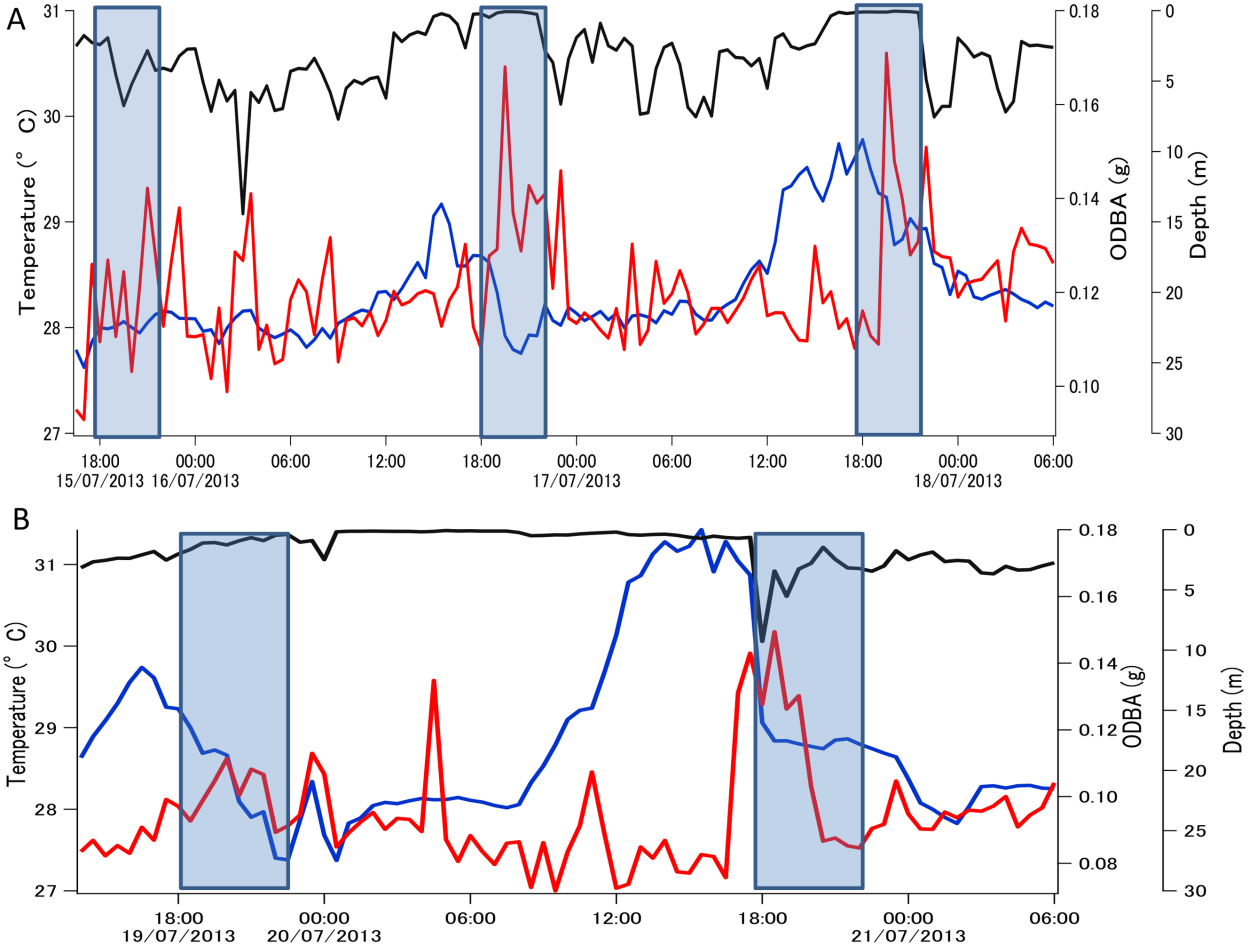
Diel changes in activity (ODBA, red line), water temperature (blue line) and swimming depth (black line) in blacktip reef sharks # 9 (A) and # 10 (B). Data are 30 minute running averages. Crepuscular periods are shaded. Note the absence of an activity peak during days without peaks in ambient temperature.

Swimming depth (from loggers) varied over the diel cycle with animals generally using shallower water at night but was primarily tidally driven, with animals swimming in very shallow water during the low tide (Table 2, S1C Table). Tides in Palmyra are semi-diurnal with cycles of approximately 6 h. During these periods of very shallow swimming during the day, pitch variability decreased (simply due to sharks swimming at the surface), but there were no obvious increases in swim speed or ODBA (S2 Fig). Video footage recorded during the morning low tide period shows that the shark was in shallow backreef and sandflat habitats. The individual frequently associated with schools of mullet (*Mugil cephalus*), other sharks and trevally, but there was no clear evidence of foraging (Fig 4, S2 Fig and S1 Video). At times the shark would swim at the surface where external temperatures increased by up to 2°C (S2 Fig and S1 Video). Two sharks showed daily differences in the extent of diel changes in ambient temperature (likely due to cloud cover or rain during the day): the evening peaks in activity were reduced or absent on days when there were minimal diel temperature peaks (Fig 5). During the seven days of deployment, the single shark fitted with a digestion logger showed clear daily peaks in motility with a small peak at dawn (approximately 6:00) and a larger one at 16:00–17:00 (Fig 3B, Table 2).

### Potential prey activity

In total, 312 focal follows were performed between 9:24 and 17:00 on the Palmyra backreefs. Foraging activity increased throughout the day with a peak in bite rates between 14:00–15:00 (quadratic regression; F_(213)_ = 51.87, R^2^ = 0.32, p < 0.001). The peak in bite rates coincided with the period when water temperatures were highest. Subsequently, bite rates slowly declined until the final observation period, limited by daylight (Fig 3C).

## Discussion

Sharks showed activity cycles that were related to both diel and tidal periods and contrary to our predictions were most active during early evening periods and ebbing high tides. While it is difficult to translate changes in ODBA to a simple biological metric (without respirometry experiments), in terrestrial endotherms (range 0.8–70 kg) an increase in ODBA of 0.2 g (the range seen in reef sharks) caused oxygen consumption to almost double [28]. While we certainly cannot extrapolate that result to sharks, it does suggest the diel rhythms in ODBA we observed were biologically meaningful. We assume these peaks in activity are related to foraging but we acknowledge that other behaviours such as predator avoidance may be responsible [34].

Why did sharks select this time period for maximal activity? Many predators have vision well suited to nocturnal conditions which gives them an advantage over their prey during low light periods. This is also true for sharks, which possess a reflective layer of cells (the tapetum lucidum) behind the retina, improving vision under low-light conditions [35]. In some active terrestrial carnivores (e.g. cheetahs), nocturnal activity is positively correlated with moonlight levels, as the predator is more likely to see and locate prey during brighter nights [36]. We found that blacktip reef sharks also increased activity during periods of high moon illumination (full moon), but this increase was also seen during periods of very low levels of moon illumination (new moon). It is more likely that these peaks in activity were related to tidal cycles rather than levels of illumination (see below).

Tidal cycles have been shown to influence movement and presumably behaviour of many shark and teleost species (e.g. [22, 23, 37, 38]). Ebbing high tides will force smaller fishes and other prey off the sandflats, which will likely increase the shark’s foraging success. Previous active tracking of blacktip reef sharks at Palmyra demonstrated that individuals maintained relatively small home ranges within which they patrolled sand-flat ledges, likely enabling them to intercept prey leaving the flats [23]. Finally, there can also be significant changes in water temperature associated with changing tides, so thermal effects may also play a role (see below, [23]). When taken together, it appears that blacktip reef shark activity is primarily driven by diel period and secondarily by tidal state.

While improved foraging may explain nocturnal and tidal activity, it still does not explain why the peak was highest in the early evening. Small fishes may compete for shelter at dusk with some individuals forced to the outer boundaries of coral heads and other structures [3]. The consumption of these individuals leads to the crepuscular peak in foraging seen in sit and wait predators. However, Holbrook and Schmitt [3] observed no crepuscular peak in activity for roaming predators such as trevally, which we might expect to be more similar in behaviour to the obligate swimming reef sharks studied here. Also, in blacktip reef sharks the peak in activity occurred just after dusk sometimes during periods of complete darkness.

Our predictions regarding the relationship between temperature and activity were only partially supported. While sharks were least active while at their warmest, they were also most active while body temperatures were warm, but cooling. Are peaks in activity related to cooling body temperatures, and if so why? Diel changes in body temperatures in blacktip reef sharks were on average 2.4°C (but up to 2.7°C), and most sharks have a Q_10_ of 2.5–3.0 (i.e. metabolic rates increase by a factor of 2.5–3 for every 10°C increase in body temperature), so metabolic rates would vary by at least ± 60% [39]. While predators can be more active when their body temperatures are high, the same applies to their prey [8, 9]. Indeed, escape behaviour has a greater scaling coefficient with temperature than attack rates, as prey have more to lose (i.e. their life) from a predator-prey interaction [9]. The herbivorous fishes at Palmyra show a peak in foraging activity when temperatures are highest. Such behaviour is common in tropical herbivorous fishes and may be due to the effects of temperature on metabolic and digestive rates and/or the effect of time on day on nutrient content of algae (which increases throughout the day, e.g. [40]). However, smaller fishes will also have lower thermal inertia and their body temperatures will decrease at a faster rate. Leopard sharks (*Triakis semifasciata*), which are similarly sized to blacktip reef sharks, have thermal body coefficients (rate of change of body temperature) of 0.0051 ± 0.001°C/min [41]. Through thermal inertia alone, shark body temperatures will take several hours to decrease. Even after an hour of cooling, sharks will still have relatively high body temperatures, while over the same time period the temperature of their smaller prey will have returned to daily low values. Hence, predator behaviour may aim to maximize foraging success based on both prey behaviour and the physiological processes that can influence behaviour of prey (i.e. metabolic rates). Further evidence that body temperature may partially drive the evening peak in activity in sharks is provided by the individuals that showed a reduced (or absent) activity peak on days when there was little temperature variability (i.e. there would be minimal cooling of body temperatures). While we cannot definitively show that cooling body temperatures drive peaks in activity, we can confirm that shark behaviour did not conform to simple ‘hunt warm, rest cold’ or ‘hunt cold, rest warm’ routines. Instead, the sharks appear to be ‘hunting and resting warm’, but they do not hunt at their *warmest*.

Digestion is also an energetically expensive process, and can continue for many hours or even days after feeding [16]. Although measurements were taken from a single animal, this is the first measurement of real-time changes in the state of digestion in a free-ranging fish. Captive experiments have shown gastric motility in blacktip reef sharks to be positively correlated with body temperature, regardless of feeding [25]. Feeding actually induces a delay in gastric contractions that can last 7–12 h (‘gastric accommodation’, [25]). The small morning peak observed here in the field may represent the end of the gastric accommodation phase, with motility levels increasing throughout the day as the body warms. Increased levels of gastric motility will likely increase gastric evacuation rates, allowing a faster return of appetite [15]. Hence, sharks may warm up during the day to increase rates of digestion and subsequent feeding rates [14]. The lag between the peaks in gastric motility and activity may represent a compromise between two energetically expensive processes: being active and digesting (similar to diving and digestion in seabirds, [30]). Similarly, eels show reduced activity during periods of increased metabolic rates associated with digestion [17]. Therefore, our hypothesis that sharks are least active while digesting is supported, and this appears to occur when the animals are at their warmest. However, the gastric data presented here is from a single shark and acceleration and digestion data were not taken from the same individual, so more work will need to be done in this area as it may represent a fruitful area of research.

While sharks may tailor their behaviour to diel changes in body temperature, it remains difficult to determine if they actively behaviourally thermoregulate. Excursions onto very shallow sandflats during low tide in the daytime caused ambient temperatures to increase by 1–4°C. We did not record temperature within specific habitats; only the water temperature experienced by the sharks, so cannot confirm active thermoregulation. However, video footage and depth sensors confirmed that sharks were swimming very close to the surface and in some cases were directly exposed to sunlight, which should also increase body warming. Female blacktip reef sharks in Australia and juvenile lemon sharks within a Bahamian nursery also appear to actively thermoregulate, selecting thermal habitats to increase body temperatures during the afternoon [24, 42].

There could be other reasons why sharks select shallow habitats during periods of low tide. They may still be thermoregulating, but for reasons associated with reproduction and gestation. Pregnant female sharks are known to aggregate and select warm shallow water during the afternoons to increase body temperature, potentially reducing gestation time of pups [24, 41]. We only tagged mature female sharks, but they did not appear to be pregnant and no shallow water aggregations are seen at Palmyra. While reproductive state (e.g. pregnancy) could explain thermoregulation, it would not explain the evening peaks in activity.

As predicted by recent foraging models, even sharks that swim continuously show behavioural routines with peaks in activity followed by ‘resting’ periods [1]. Selecting when this peak in activity occurs will be of critical importance for the fitness of the animal. While we clearly show when sharks choose to be most active, disentangling the contributing factors is difficult and several of these factors may not be mutually exclusive. For example, sharks may demonstrate crepuscular peaks because these represent a period when light levels are lower, but body temperatures are still elevated but cooling. However, our results do suggest that blacktip sharks at Palmyra attempt to maximize ingestion rates over efficiency of energy use. ‘Active while cooling’ may be a more common behaviour than previously recognized for ectothermic marine predators in tropical environments [2].

The implications of predator activity patterns and the effects of temperature on predator-prey relationships are profound as these will flow from the individual to the population and are critical to interpreting or predicting the impacts of climate change [8, 43]. The loss of large predators at tropical islands is believed to cause a switch in diel behaviour of prey fishes (i.e., nocturnal species become diurnal) related to the predators activity patterns [18]. High-resolution measurements of predator and prey activity are required to advance and test these ideas. Ultimately, we must determine how factors such as hunting success, physiological performance, and social associations guide the myriad of decisions animals make and how temperature may influence them.

## Supporting Information

S1 Fig Generalized Additive Model results showing effect of time of day on acceleration, swimming depth and body temperature for individual blacktip reef sharks. Fig A Diel changes in ODBA. Fig B diel changes in ODBA standardized raw data. Fig C tidal effects on ODBA. Fig D Distribution of residuals around GAMM splines for telemetry data. Fig E distribution of residuals around GAM splines for diel changes in body temperature in four individuals sharks. Fig F raw data showing diel changes in body temperature from blacktip reef sharks.
(DOCX)

S2 Fig A Raw data showing change in swimming depth, speed, water temperature and pitch of blacktip reef shark #9. (DOCX)

S1 Table Generalized Additive Model results for individual sharks. Table A predictors of acceleration, body temperature, and swimming depth (from transmitters), Table B predictors of ODBA and swim speed from data-loggers). Table C predictors of swimming depth (from data-loggers). Table D average body temperatures and swimming depths.
(DOCX)

S1 Video Footage from blacktip reef shark. Shark investigate a school of mullet with no obvious response.
(WMV)
