# Supplementary material for: Drivers of Daily Routines in an Ectothermic Marine Predator: Hunt Warm, Rest Warmer?
Source: PLoS One. 2015 Jun 10;10(6):e0127807. doi: 10.1371/journal.pone.0127807 (PMC4489509; doi:10.1371/journal.pone.0127807)
Supplement: S1 Fig — Fig A Diel changes in ODBA. Fig B diel changes in ODBA standardized raw data. Fig C tidal effects on ODBA. Fig D Distribution of residuals around GAMM splines for telemetry data. Fig E distribution of residuals around GAM splines for diel changes in body temperature in four individuals sharks. Fig F raw data showing diel changes in body temperature from blacktip reef sharks. (DOCX) [file pone.0127807.s001.docx]

S2. Behavioural routines in individual sharks and distribution of residuals

Fig. A. Individual plots of GAM splines displaying relationship between ODBA (log scale), time of day and ambient water temperature (blue points). ODBA spline is estimated from one-minute means and dashed lines are 95 % confidence intervals. Y-axes are standardized residuals (for ODBA). A-D are for sharks # 7-10.

Fig. B. ODBA standardized raw data for individual sharks (each colour is a different shark) plotted around the GAMM spline (black line mean, dashed line 95 % confidence interval).

Fig. C. Individual plots of GAMM splines displaying relationship between ODBA (log scale), time of day and tidal height (blue points). ODBA spline is estimated from a five-minute mean and dashed lines are 95 % confidence intervals. Y-axes are standardized residuals (for ODBA). A-D are for shark # 7-10.


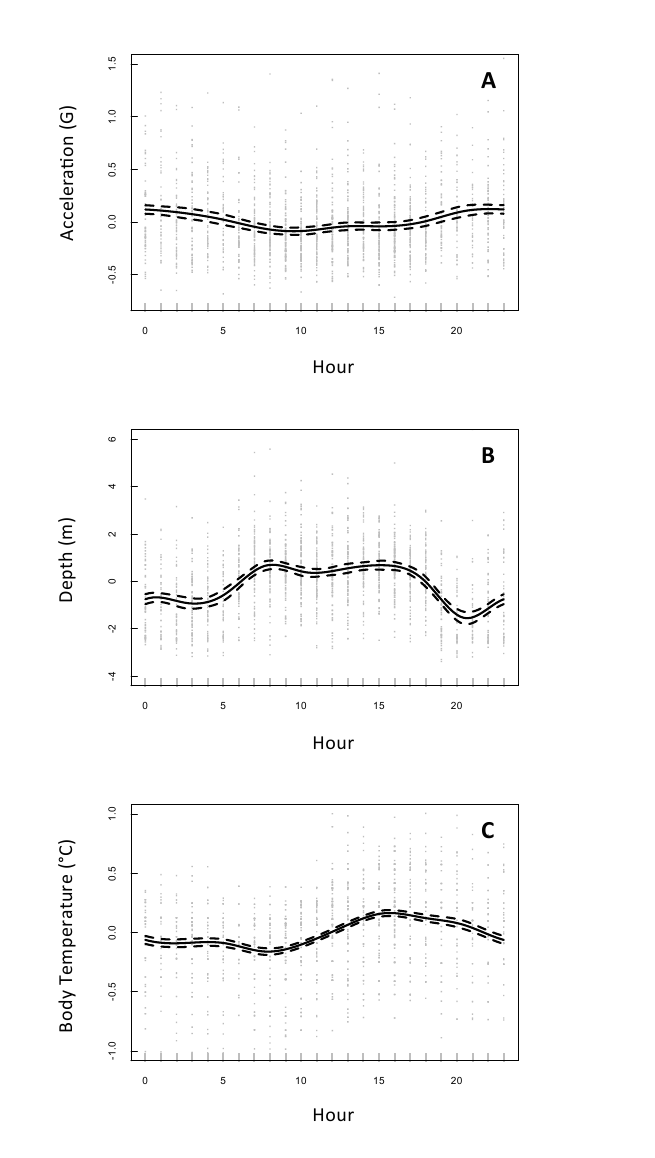


Fig. D. Distribution of residuals around GAMM splines for telemetry data.


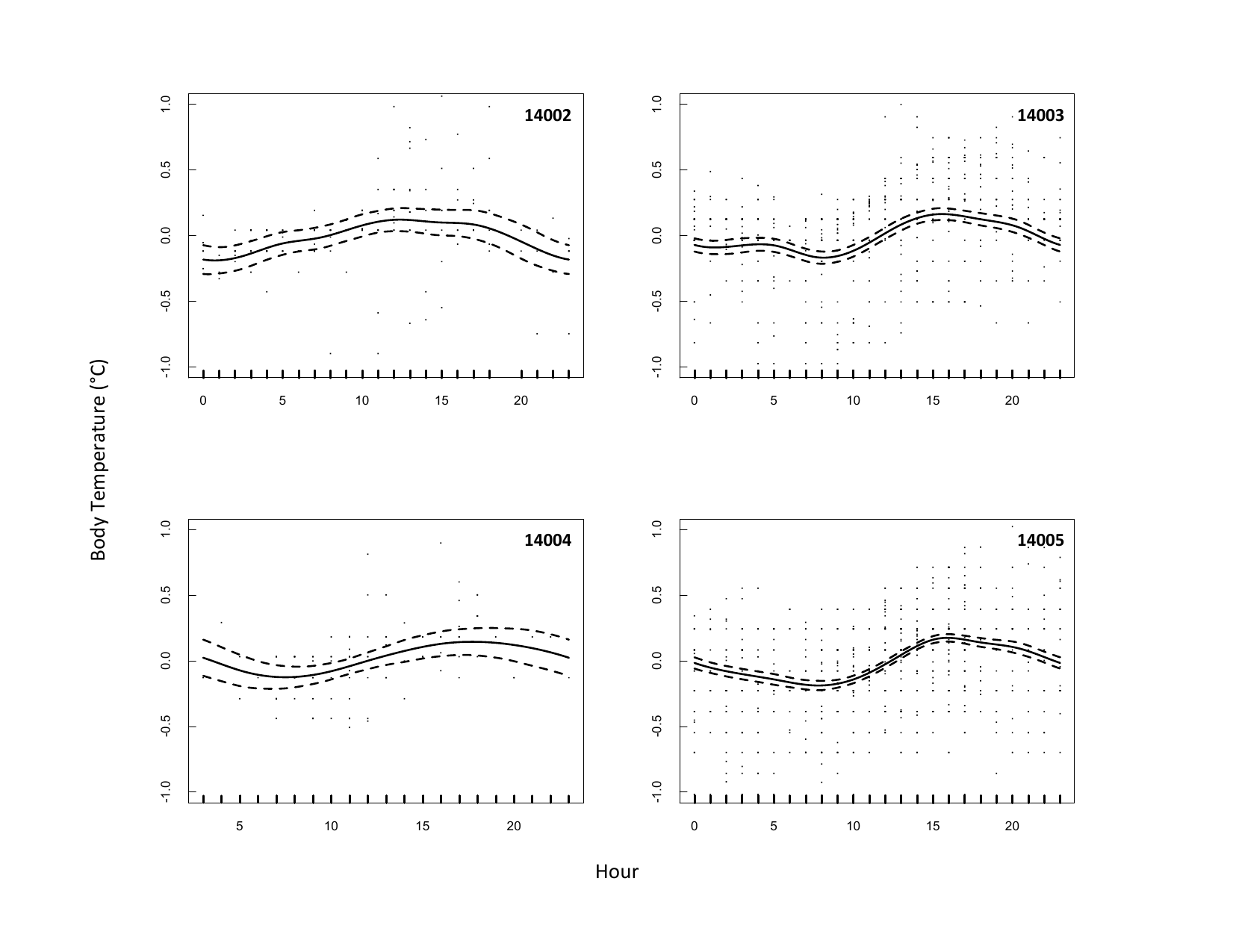


Fig. E. Distribution of residuals around GAM splines for diel changes in body temperature, from four individual blacktip reef sharks.

Fig F. Diel changes in body temperature (from transmitters) for 4 blacktip reef sharks at Palmyra atoll.
