# Supplementary material for: Drivers of Daily Routines in an Ectothermic Marine Predator: Hunt Warm, Rest Warmer?
Source: PLoS One. 2015 Jun 10;10(6):e0127807. doi: 10.1371/journal.pone.0127807 (PMC4489509; doi:10.1371/journal.pone.0127807)
Supplement: S2 Fig — (DOCX) [file pone.0127807.s002.docx]

S3. Raw behavioural data of low-tide shallow water swimming

Fig. A. Shallow water swimming in shark #9. During very shallow swimming there is a decrease in pitch variability and an increase in ambient temperature, but no increase in swim speed. Note, although at the surface, based on video footage, the swim speed sensor was rarely above the water-line (which would have influenced the sensor readings).
