# Supplementary material for: Drivers of Daily Routines in an Ectothermic Marine Predator: Hunt Warm, Rest Warmer?
Source: PLoS One. 2015 Jun 10;10(6):e0127807. doi: 10.1371/journal.pone.0127807 (PMC4489509; doi:10.1371/journal.pone.0127807)
Supplement: S1 Table — Table A predictors of acceleration, body temperature, and swimming depth (from transmitters), Table B predictors of ODBA and swim speed from data-loggers). Table C predictors of swimming depth (from data-loggers). Table D average body temperatures and swimming depths. (DOCX) [file pone.0127807.s003.docx]

S1. GAM results for telemetry and data-logger deployments

| **Model and terms x** | ***n*** | **Smoother** | **Degrees of**  **freedom** | ***F*-statistic** | ***P*** | ***r^2^*** |
| --- | --- | --- | --- | --- | --- | --- |
|  |  |  |  |  |  |  |
| Body Temperature shark 3 | 1 |  |  |  |  | 0.14 |
| s(Hour) |  | cyclic | 3.68 | 2.07 | <0.001 |  |
| Body Temperature shark 4 | 1 |  |  |  |  | 0.14 |
| s(Hour) |  | cyclic | 6.61 | 12.3 | <0.001 |  |
| Body Temperature shark 5 | 1 |  |  |  |  | 0.19 |
| s(Hour) |  | cyclic | 2.50 | 1.48 | 0.002 |  |
| Body Temperature shark 6 | 1 |  |  |  |  | 0.09 |
| s(Hour) |  | cyclic | 6.50 | 29.43 | <0.001 |  |
| Depth shark 1 | 1 |  |  |  |  | 0.25 |
| s(Hour) |  | cyclic | 6.20 | 15.37 | <0.001 |  |
| Depth shark 2 | 1 |  |  |  |  | 0.24 |
| s(Hour) |  | cyclic | 6.19 | 6.81 | <0.001 |  |
| Depth shark 3 | 1 |  |  |  |  | 0.11 |
| s(Hour) |  | cyclic | 4.35 | 1.8 | 0.007 |  |
| Depth shark 4 | 1 |  |  |  |  | 0.32 |
| s(Hour) |  | cyclic | 7.22 | 26.41 | <0.001 |  |
| Depth shark 5 | 1 |  |  |  |  | 0.41 |
| s(Hour) |  | cyclic | 6.53 | 21.67 | <0.001 |  |
| Depth shark 6 | 1 |  |  |  |  | <0.01 |
| s(Hour) |  | cyclic | <0.01 | <0.01 | 0.68 |  |
| Acceleration shark 1 | 1 |  |  |  |  | 0.23 |
| s(Hour) |  | cyclic | 2.07 | 1.07 | 0.006 |  |
| s(Lunar fraction illuminated) |  | cyclic | 6.39 | 11.62 | <0.001 |  |
| factor(Month) |  | -- | -- | -- | -- |  |
| Acceleration shark 2 | 1 |  |  |  |  | 0.01 |
| s(Hour) |  | cyclic | 0.41 | 0.06 | 0.31 |  |
| s(Lunar fraction illuminated) |  | cyclic | <0.01 | <0.01 | 0.714 |  |
| Acceleration shark 3 | 1 |  |  |  |  | <0.01 |
| s(Hour) |  | cyclic | <0.01 | <0.01 | 0.50 |  |
| s(Lunar fraction illuminated) |  | cyclic | <0.01 | <0.01 | 0.46 |  |
| factor(Month) |  | -- | -- | -- | -- |  |
| Acceleration shark 4 | 1 |  |  |  |  | 0.11 |
| s(Hour) |  | cyclic | 5.00 | 6.99 | <0.001 |  |
| s(Lunar fraction illuminated) |  | cyclic | <0.01 | <0.01 | 0.54 |  |
| factor(Month) |  | -- | -- | -- | -- |  |
| Acceleration shark 5 | 1 |  |  |  |  | <0.01 |
| s(Hour) |  | cyclic | 1.21 | 0.29 | 0.14 |  |
| s(Lunar fraction illuminated) |  | cyclic | <0.01 | <0.01 | 0.46 |  |
| factor(Month) |  | -- | -- | -- | -- |  |
| Acceleration shark 6 | 1 |  |  |  |  | <0.01 |
| s(Hour) |  | cyclic | <0.01 | <0.01 | 0.30 |  |
| s(Lunar fraction illuminated) |  | cyclic | <0.01 | <0.01 | 0.56 |  |
| factor(Month) |  | -- | -- | -- | -- |  |

Table A. GAM results showing effects of time of day and lunar cycle on body temperature, acceleration and swimming depth (all from transmitters) for individual sharks.

| **Model and terms** | ***n*** | **Smoother** | **Degrees of**  **freedom** | ***F*-statistic** | ***P*** | ***r^2^*** |
| --- | --- | --- | --- | --- | --- | --- |
|  |  |  |  |  |  |  |
| ln OBDA shark 7 | 1 |  |  |  |  | 0.15 |
| s(Hour) |  | cyclic | 2.71 | 2.64 | <0.001 |  |
| s(Tide) |  | cubic | 3.71 | 20.96 | <0.001 |  |
| s(Depth) |  | cubic | 8.05 | 51.11 | <0.001 |  |
| s(Ambient Temperature) |  | cubic | 5.82 | 9.51 | <0.001 |  |
| ln OBDA shark 8 | 1 |  |  |  |  | 0.19 |
| s(Hour) |  | cyclic | 6.96 | 28.91 | <0.001 |  |
| s(Tide) |  | cubic | 3.61 | 19.03 | <0.001 |  |
| s(Depth) |  | cubic | 7.29 | 15.32 | <0.001 |  |
| s(Ambient Temperature) |  | cubic | 8.09 | 7.69 | <0.001 |  |
| ln OBDA shark 9 | 1 |  |  |  |  | 0.10 |
| s(Hour) |  | cyclic | 7.20 | 11.37 | <0.001 |  |
| s(Tide) |  | cubic | 1.00 | 1.37 | 0.242 |  |
| s(Depth) |  | cubic | 1.00 | 11.70 | <0.001 |  |
| s(Ambient Temperature) |  | cubic | 4.44 | 7.21 | <0.001 |  |
| ln OBDA shark 10 | 1 |  |  |  |  | 0.16 |
| s(Hour) |  | cyclic | 3.60 | 4.72 | <0.001 |  |
| s(Tide) |  | cubic | 3.23 | 5.71 | <0.001 |  |
| s(Depth) |  | cubic | 1.86 | 4.59 | 0.012 |  |
| s(Ambient Temperature) |  | cubic | 4.70 | 1.85 | 0.104 |  |
| Speed shark 9 | 1 |  |  |  |  | 0.22 |
| s(Hour) |  | cyclic | 5.74 | 7.31 | <0.001 |  |
| s(Tide) |  | cubic | 3.31 | 5.51 | <0.001 |  |
| s(Depth) |  | cubic | 8.93 | 28.31 | <0.001 |  |
| Speed shark 10 | 1 |  |  |  |  | 0.21 |
| s(Hour) |  | cyclic | 4.04 | 18.38 | <0.001 |  |
| s(Tide) |  | cubic | 5.03 | 3.67 | 0.003 |  |
| s(Depth) |  | cubic | 1.00 | 2221.15 | <0.001 |  |

Table B. GAM results for the effects of time of day, tidal height, swimming depth and ambient temperature on ODBA (log transformed) and swim speed from individual blacktip reef sharks (all data obtained using data-loggers).

| **Model and terms** | ***n*** | **Smoother** | **Degrees of**  **freedom** | ***F*-statistic** | ***P*** | ***r2*** |
| --- | --- | --- | --- | --- | --- | --- |
|  |  |  |  |  |  |  |
| Depth shark 7 | 1 |  |  |  |  | 0.18 |
| s(Time of day) |  | cyclic | 6.53 | 6.12 | <0.001 |  |
| s(Tide) |  | cubic | 3.05 | 11.80 | <0.001 |  |
| Depth shark 8 | 1 |  |  |  |  | 0.04 |
| s(Time of day) |  | cyclic | 4.50 | 2.33 | 0.047 |  |
| s(Tide) |  | cubic | 7.55 | 7.74 | <0.001 |  |
| Depth shark 9 | 1 |  |  |  |  | 0.11 |
| s(Time of day) |  | cyclic | 2.35 | 3.49 | 0.023 |  |
| s(Tide) |  | cubic | 7.24 | 8.78 | <0.001 |  |
| Depth shark 10 | 1 |  |  |  |  | 0.01 |
| s(Time of day) |  | cyclic | 2.44 | 3.6 | 0.020 |  |
| s(Tide) |  | cubic | 6.68 | 7.64 | <0.001 |  |

Table C. GAM results for effects of time of day and tidal height on swimming depth (from data-loggers) in individual sharks.

| Shark # | Body temperature (°C) |  |  | Depth (m) |  |  |
| --- | --- | --- | --- | --- | --- | --- |
|  | Mean | Min | Max | Mean | Min | Max |
| 3 | 29.04 | 28.26 | 30.46 | 3.09 | 0 | 13.65 |
| 4 | 29.00 | 28.11 | 30.62 | 2.41 | 0 | 6.4 |
| 5 | 29.05 | 28.26 | 30.93 | 2.59 | 0 | 7.60 |
| 6 | 28.77 | 28.58 | 30.77 | 1.58 | 0 | 14.55 |

Table D. Average body temperatures and swimming depths for four blacktip reef sharks fitted with acoustic transmitters.
